# Epigenetic marker of telomeric age is associated with exacerbations and hospitalizations in chronic obstructive pulmonary disease

**DOI:** 10.1186/s12931-021-01911-9

**Published:** 2021-12-22

**Authors:** Ana I. Hernández Cordero, Chen Xi Yang, Xuan Li, Stephen Milne, Virginia Chen, Zsuzsanna Hollander, Raymond Ng, Gerard J. Criner, Prescott G. Woodruff, Stephen C. Lazarus, John E. Connett, MeiLan K. Han, Fernando J. Martinez, Robert M. Reed, S. F. Paul Man, Janice M. Leung, Don D. Sin

**Affiliations:** 1 Centre for Heart Lung Innovation, St. Paul’s Hospital and University of British Columbia, Room 166-1081 Burrard St, Vancouver, BC V6Z 1Y6 Canada; 2 Division of Respiratory Medicine, Department of Medicine, University of British Columbia, Vancouver, BC Canada; 3 Sydney Medical School, Faculty of Medicine and Health Sciences, University of Sydney, Sydney, NSW Australia; 4 PROOF Centre of Excellence, St. Paul’s Hospital, Vancouver, BC Canada; 5 Department of Thoracic Medicine and Surgery, Temple University, Philadelphia, PA USA; 6 Department of Medicine, University of California San Francisco, San Francisco, CA USA; 7 School of Public Health, University of Minnesota, Minneapolis, MN USA; 8 Department of Internal Medicine, University of Michigan, Ann Arbor, MI USA; 9 Joan and Sanford I. Weill Department of Medicine, Weill Cornell Medical College, Cornell University, New York, NY USA; 10 Division of Pulmonary and Critical Care Medicine, University of Maryland School of Medicine, Baltimore, MD USA

**Keywords:** COPD, Telomeres, AECOPD, Epigenetics, DNA methylation

## Abstract

**Background:**

Chronic obstructive pulmonary disease (COPD) is an age-related condition that has been associated with early telomere attrition; the clinical implications of telomere shortening in COPD are not well known. In this study we aimed to determine the relationship of the epigenetic regulation of telomeric length in peripheral blood with the risk of exacerbations and hospitalization in patients with COPD.

**Methods:**

Blood DNA methylation profiles were obtained from 292 patients with COPD enrolled in the placebo arm of the Macrolide Azithromycin to Prevent Rapid Worsening of Symptoms Associated with Chronic Obstructive Pulmonary Disease (MACRO) Study and who were followed for 1-year. We calculated telomere length based on DNA methylation markers (DNAmTL) and related this biomarker to the risk of exacerbation and hospitalization and health status (St. George Respiratory Questionnaire [SGRQ]) score over time using a Cox proportional hazards model. We also used linear models to investigate the associations of DNAmTL with the rates of exacerbation and hospitalization (adjusted for chronological age, lung function, race, sex, smoking, body mass index and cell composition).

**Results:**

Participants with short DNAmTL demonstrated increased risk of exacerbation (*P* = *0.02*) and hospitalization (*P* = *0.03*) compared to those with longer DNAmTL. DNAmTL age acceleration was associated with higher rates of exacerbation (*P* = *1.35* × *10*^*–04*^) and hospitalization (*P* = *5.21* × *10*^*–03*^) and poor health status (lower SGRQ scores) independent of chronological age (*P* = *0.03*).

**Conclusion:**

Telomeric age based on blood DNA methylation is associated with COPD exacerbation and hospitalization and thus a promising biomarker for poor outcomes in COPD.

**Supplementary Information:**

The online version contains supplementary material available at 10.1186/s12931-021-01911-9.

## Background

Chronic obstructive pulmonary disease (COPD) is characterized by persistent airway inflammation and airflow limitation. COPD is an age-related condition that is associated with cellular senescence [1]. One form of early ageing is replicative senescence, which is closely linked with accelerated telomere attrition. Telomeres are repetitive DNA sequences at the end of chromosomes. Their primary function is to protect the chromosome from damage and prevent premature cell death or abnormal cell proliferation. With each cell cycle, telomeres shorten until a critical length is reached just prior to a permanent cell cycle arrest [2]. Exposure to noxious particles such as tobacco smoke, or enhanced inflammatory process can accelerate telomere shortening [3] and induce cellular senescence [4]. It is well known that short telomeres are associated with increased risk of COPD and decreased lung function [5]. However the clinical consequences of telomere shortening and cellular senescence in COPD are not fully understood.

The traditional techniques that measure telomere length, such as Quantitative Polymerase Chain Reaction (Q-PCR), have technical limitations, including inconsistent reproducibility between laboratories, amongst others [6]. Even the gold standard measurement for telomeres, Terminal Restriction Fragment (TRF) analysis, has its limitations. While TRF captures a range of telomere sizes, the shortest telomeres are not visualized [6, 7]. These are mainly a result of replicative senescence, thus limiting TRF from accurately reflecting cellular replication. Furthermore, telomeres can vary between individuals from birth [8], and specific haplotypes in the telomerase gene are associated with longer telomeres [9]. Thus, telomere shortening based on measurements of absolute and relative telomeric length is likely confounded by the effect of telomerase activity.

Telomere shortening is associated with epigenetic activity characterized by DNA methylation [10, 11]. DNA methylation is determined by the dynamic addition or removal of a methyl group from a cytosine–guanine residue. DNA methylation is a well-established marker of biological age [12, 13] that can be altered by disease, chronic infections or environmental factors. Thus, the examination of peripheral blood DNA methylation profiles can provide insights into the mechanisms that contribute to cellular ageing in COPD. To date, multiple epigenetic “clocks” have been developed to determine biological age of individuals and these have been shown to predict mortality in the general population [12, 13]. To investigate the epigenetic regulation of telomere length, a DNA methylation marker of telomeric age has been developed and has been shown to have stronger correlation with chronological age than telomere length measurements themselves [11]; however, its relationship with various COPD outcomes is unknown.

Here, we hypothesized that in COPD patients, advanced epigenetic telomeric age is associated with increased risk of exacerbations and poor health outcomes. Our primary aim was to investigate the clinical impact of telomeric ageing in COPD by applying a DNA methylation blood biomarker of telomere length (DNAmTL).

## Methods

### Study cohort

For the present analyses we utilized data from the Macrolide Azithromycin to Prevent Rapid Worsening of Symptoms Associated with Chronic Obstructive Pulmonary Disease (MACRO) Study [14]. This study was a randomized controlled trial that was conducted between 2006 and 2010 (ClinicalTrials.gov identifier: NCT00325897). Its design, as well as its inclusion and exclusion criteria, have been previously described [14–16]. Briefly, MACRO included participants with a clinical diagnosis of COPD as defined by the Global initiative for chronic Obstructive Lung Disease (GOLD) [4]. Participants were 40 years of age or older, and were cigarette users (10 pack-years or more; either currently or in the past). In addition, the participants had a history of supplemental oxygen use or had received a course of systemic corticosteroids or antibiotics within the year prior to study entry but not within 4 weeks of enrollment. Participants had to be clinically stable at the time of recruitment without a prior history of asthma, bronchiectasis, or chronic renal or hepatic insufficiency. Blood samples were collected at baseline using a standard venipuncture protocol. Patients were randomly assigned to 250 mg/day of azithromycin or a matching placebo in a 1:1 ratio. The participants were followed for 12 months. To remove the confounding effects of azithromycin on exacerbation rates, we used data only from participants in the placebo arm (n = 292) of MACRO, who provided blood and consented to genetic studies (Additional file 1). This subset of patients had similar lung function, body mass index (BMI), and gender distribution as participants in whom DNA materials were not collected (Additional file 2).

### Health outcomes

Health outcomes recorded in MACRO included exacerbations, hospitalizations, lung function (as expressed by forced expiratory volume in 1 s [FEV_1_] and forced vital capacity [FVC]) and health status (as measured by the St. George’s Respiratory Questionnaire [SGRQ]) [14, 15]. Acute COPD exacerbations (AECOPDs) were defined as events that resulted in a significant increase in patient symptoms leading to modification of COPD therapy. AECOPDs were graded in terms of severity using the following scale: (i) mild: treatment conducted at home (with or without contacting a health care provider); (ii) moderate: treatment requiring a visit to the emergency room or the institution of systemic corticosteroids and/or antibiotics; (iii) severe: treatment requiring hospitalization; and (iv) very severe: treatment requiring hospitalization and ventilatory support. For the present study, we analyzed the data based on total counts of AECOPD (regardless of severity), and in subsequent analyses focused on mild and moderate to severe AECOPDs.

### DNA methylation profiling

DNA was extracted from blood samples and processed according to a previously described pipeline [17, 18]. Unmethylated cytosine residues in the DNA extracts were converted to uracil. The Illumina HumanMethylation450 microarray was used to profile methylation sites (CpGs probes). The beta-value for each CpG probe was calculated as the ratio of methylation probe intensity to the overall intensity ranging from 0 (fully unmethylated) to 1 (fully methylated). CpG probes with a detection *P* > *1* × *10*^*–10*^ in at least 5% of the samples, non-CG probes, and XY-chromosome probes were removed. We used Normal-exponential out-of-band [19], Beta-Mixture Quantile Normalization [20] and ComBat [21] methods for background correction, normalization, and batch correction, respectively. At the end of this process, we were able to retain 467,368 CpG probes for downstream analyses.

### Epigenetic marker of telomere length

We estimated DNA methylation measurement of telomere length (DNAmTL) for each sample using a method described by Horvath et al. [11]. DNAmTL was derived from 140 methylation sites (CpGs) that were highly associated with leukocyte telomere length. We determined the association between DNAmTL and the following outcome variables: number of AECOPDs (total, mild, and moderate to severe), rate of exacerbations, hospitalization, number of hospitalizations, rate of hospitalization, and SGRQ (measured on the same visit as the blood draw). Absolute telomere length (aTL) was previously measured in peripheral blood of the MACRO participants by using quantified polymerase chain reaction [16]. We thus compared aTL and DNAmTL by determining their correlation using the statistical software R.

### Statistical analyses

#### Principal component analysis

A principal component analysis (PCA) based on cell proportions (D8T, CD4T, NK, B, Monocytes and Granulocytes) was conducted. DNA methylation varies depending on tissue and cell type [22]; therefore PCs that explained more that 5% of the variance were used to adjust the statistical models (PC1 to PC5). PCA was performed using the “prcomp” function implemented in the “stats” R package [23].

#### Cox proportional-hazards regression analysis

We investigated the effect of DNAmTL age acceleration on exacerbation and hospitalization over time. DNAmTL age acceleration was defined as the residuals obtained from the regression of DNAmTL on chronological age. Negative residuals indicate shorter DNAmTL than expected based on age (and positive residuals indicate the opposite). We calculated DNAmTL age acceleration for each sample and adjusted this value for potential confounders including chronological age, FEV_1_% predicted (FEV_1_%), race, sex, smoking status, body mass index (BMI), number of months in the study, and the first 5 PCs. We ranked the participants in our cohort based on the residuals, which resulted in three groups: (i) participants with DNAmTL residual values that were in the 25th percentile (short DNAmTL); (ii) participants with DNAmTL residuals between the 25th and 75th percentiles (intermediate DNAmTL); and (iii) participants with DNAmTL residuals that were in the top 75th percentile (long DNAmTL). These categories were used to assess the probability of exacerbation and hospitalization over time using Cox regression models.

For the Cox analyses, we used (1) time to 1st AECOPD (regardless of severity); (2) time to 1st mild AECOPD; (3) time to 1st moderate to severe AECOPD; and (4) time to 1st hospitalization. Significant effects were defined using a threshold of *P* < *0.05* (likelihood ratio test). Assumption of proportional hazards for all analyses was tested using the survival R package and this assumption was met (AECOPD *P* = *0.48*, mild AECOPD *P* = *0.11*, moderate to severe AECOPD *P* = *0.49,* hospitalization *P* = *0.97*)*.* Cox analyses were performed using the “survival” package implemented in R [24].

DNAmTL and clinical features. We used a linear model (lm) to test the association of DNAmTL with exacerbation (yes/no), number of exacerbations (total, mild, and moderate–severe AECOPD), and SGRQ score. The final lm is shown below:

$$DNAmTL \sim Age +\left(variable\, of\, interest\right)+ FEV1\%pre + race + sex + smoking + BMI+ \left(number \,of\, months\, in\, the\, study\right)+(PC1\, to\, PC5)$$

A Jonckheere–Terpstra test was used to determined significant trends across DNAmTL groups for the number of AECOPDs. Significant trends were set at *P* < *0.05*. This test was executed using the “DescTools” implemented in R statistical [23]. In addition, we explored the effect of inhaled corticosteroids (ICS), long-acting muscarinic antagonists (LAMA) and long-acting beta-2 agonists (LABA) at baseline on DNAmTL, using the linear model described above.

We also tested the association between DNAmTL and the rate of exacerbation (total, mild, and moderate to severe AECOPD) and hospitalization, which was expressed as number of exacerbations or hospitalizations per year. The number of months in the study were converted to years by dividing the number of months that each participant remained in the study by 12. For these two variables we used the following lm:

$$DNAmTL \sim Age +\left(Exacerbation\, or\, hospitalization rate\right)+ FEV1\%pre + race + sex + smoking + BMI+(PC1\, to \,PC5)$$

We defined significant associations based on *P* < *0.05* for continuous variables. The P-values that corresponded to categorical variables with more than 2 levels were adjusted using a Tukey’s method for multiple comparison. Lm and P-value adjustments were performed using the R statistical software [23].

## Results

### Study cohort overview

The baseline characteristics of the study cohort are shown in Table 1. A majority of the participants experienced at least one AECOPD during follow-up (n = 211). No significant differences in age, BMI, lung function or use of inhaled corticosteroids at baseline were found between the two groups (Table 1). However, male sex was a predictor of exacerbation (*P* = *0.001*), in which men were less likely to experience exacerbations during follow-up compared to women. Lung function was lower in the subset of patients who experienced a hospitalization for AECOPD (*P* = *0.01*).

**Table 1:** Study cohort baseline characteristics

	All	Exacerbation during follow-up		*P
		No	Yes	
n	292	81	211	–
Age, year	67 ± 8	67 ± 9	67 ± 8	0.97
Males, No. (%)	165 (57)	58 (72)	107 (51)	0.001
Non-Hispanic whites, No. (%)	246 (84)	65 (80)	179 (85)	0.42
Current smokers, No. (%)	53 (18)	16 (20)	37 (18)	0.74
BMI, kg/m^2^	27 ± 6	27 ± 6	26 ± 6	0.84
Hospitalizations, No. (%)	68 (23)	–	68 (32)	–
Post-bronchodilator FVC, L	2.52 ± 0.80	2.60 ± 0.73	2.50 ± 0.83	0.23
Post-bronchodilator FVC, % of predicted	69.53 ± 17.35	69.56 ± 16.43	69.51 ± 17.73	0.75
Post-bronchodilator FEV_1_, L	1.07 ± 0.49	1.09 ± 0.47	1.07 ± 0.49	0.72
Post-bronchodilator FEV_1_, % of predicted	39.3 ± 15.62	38.96 ± 15.56	39.49 ± 15.68	0.70
FEV_1_/FVC, %	42.29 ± 12.05	41.45 ± 11.45	42.31 ± 12.28	0.41
Inhaled corticosteroid use (ICS), No. (%)	230 (79)	65 (80)	165 (78)	0.75
ICS/LABA, No. (%)	148 (77)	44 (54)	104 (49)	0.51

Continuous variables are described with the mean ± SD

^*^Differences between the groups’ demographic characteristics were tested using a Wilcoxon test for continuous variables, and a Fisher’s Exact test for count variables. “Post” refers to spirometry tests after bronchodilator use. Denominators used for the percentages (%) correspond to the total number of participants in each group

*BMI* body mass index, *FEV1* forced expiratory volume in 1 s, *FVC* forced vital capacity, *ICS* inhaled corticosteroids, *LABA* long-acting beta-2 agonists

### Epigenetic marker of telomere length is associated with COPD exacerbations

**Fig. 1:**
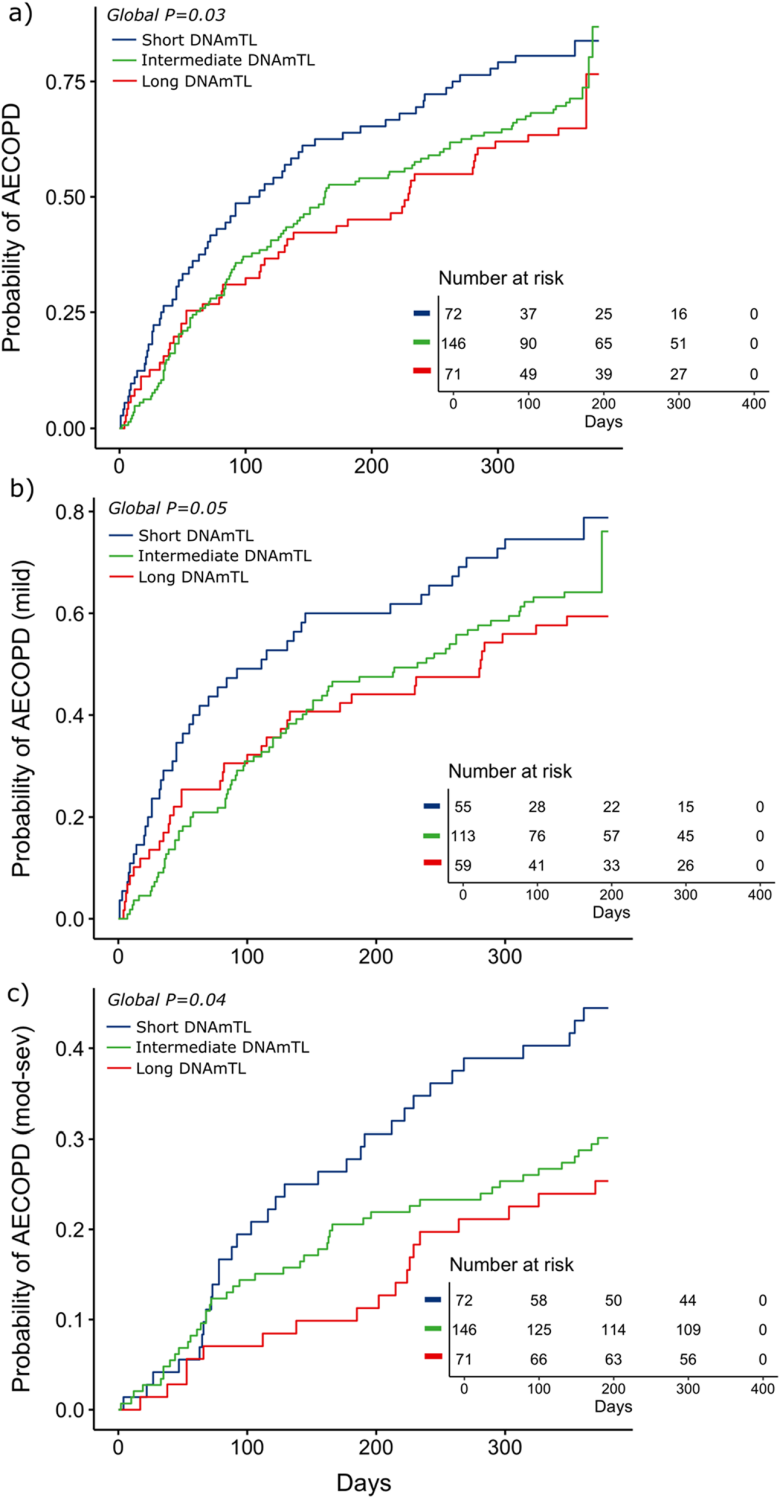
Kaplan–Meier curve for exacerbation and DNAmTL age acceleration. Association between the epigenetic measurement of telomere length (DNAmTL) and probability of exacerbation (y-axis) over time (x-axis). **a** Corresponds to the Kaplan–Meier curve for acute COPD exacerbations (AECOPD) regardless of severity, while (**b**) and (**c**) show the probability of mild and moderate to severe AECOPD over time, respectively. The group with short DNAmTL corresponds to the description “DNAmTL residuals < − 0.12 or 25th percentile” (blue line), in which DNAmTL residuals were derived from the regression of DNAmTL on chronological age adjusted for sex, body mass index, smoking status and the first five principal components of blood cell proportions. Long DNAmTL corresponds to the description “DNAmTL residuals > 0.13 or 75th percentile” (red line). The description “−12 = < DNAmTL residuals > = 0.13” (green line) corresponds to the group intermediate DNAmTL. Number of patients at risk at each timepoint are provided inside the plots. P-values were generated from a likelihood ratio test based on the global test (Cox analysis)

DNAmTL was significantly correlated with chronological age (R = − 0.56, *P* = *5.54* × *10*^*–26*^) (Additional file 3) and had a positive, albeit modest, correlation with aTL (R = 0.21, *P* = *6* × *10*^*–04*^). Before any adjustments, DNAmTL was associated with increased risk of AECOPD (*P* = *0.02*). Based on a Cox regression analysis, DNAmTL age acceleration was associated with a higher probability of exacerbation over time, regardless of its severity (Global test *P* = *0.03*) (Fig. 1a). Likewise, DNAmTL age acceleration was associated with increased probability of moderate to severe exacerbation (Global test *P* = *0.04*) (Fig. 1b). Although the Cox’s global test for the probability of mild exacerbations was borderline significant (Global test *P* = *0.05*), the comparison between the short and long DNAmTL showed that short DNAmTL was associated with a greater probability of mild AECOPD over time (*P* = *0.03*) compared to long DNAmTL. Our findings show that while individuals with short telomere length demonstrated an increased probability of exacerbation compared to those with intermediate or long telomeres, there was no significant difference observed between individuals with intermediate and long telomeres (Additional file 4).

**Fig. 2:**
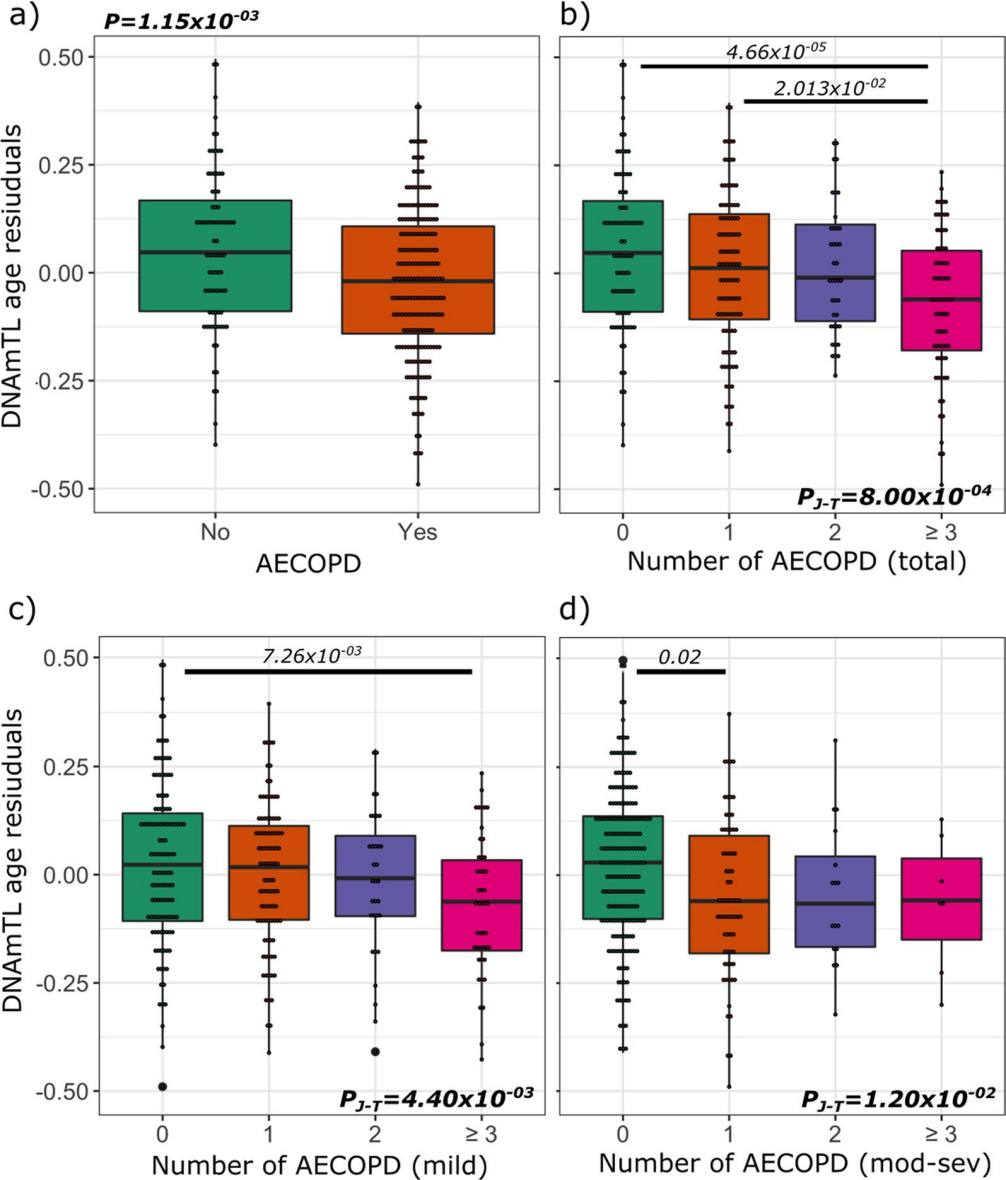
Relationship between exacerbation and DNAmTL age acceleration. Boxplots show the DNAmTL age residuals plotted against acute COPD exacerbations (AECOPDs) (**a**), number of total (**b**), mild (**c**) and moderate to severe (**d**) AECOPDs. P values at the top of (**a**), (**b**) and (**c**) correspond to the P-values adjusted for multiple comparison based on the Tukey method. The beginning and the end of the horizontal bars represent the pairwise comparison that corresponds to each P value. DNAm are residuals, which were obtained from the regression of DNAmTL on chronological age adjusted for sex, body mass index, smoking status and the first five principal components of blood cell proportions. Negative values represent age acceleration. P-value at the right corner of panel (**b**), (**c**) and (**d**) corresponds to the Jonckheere–Terpstra trend test

Participants who experienced an AECOPD at follow-up demonstrated shorter DNAmTL than expected based on chronological age (Fig. 2a). Participants who experienced three or more AECOPDs during follow-up demonstrated DNAmTL accelerated ageing compared to those who did not experience any or only one AECOPD, and there was a significant trend across DNAmTL groups (*P* = *8* × *10*^*–04*^) (Fig. 2b). A similar relationship was also observed in patients with 3 or more mild exacerbations (Fig. 2c), while DNAmTL accelerated ageing was associated with one moderate to severe exacerbation (Fig. 2d). Rate of exacerbation was associated with accelerated ageing in which higher rates of AECOPD were linked to shorter DNAmTL than expected based on chronological age. We explored the potential effect that medications might have on DNAmTL, and found that overall ICS (*P* = *0.96*), LABA (*P* = *0.52*), or LAMA (*P* = *0.16*) had no significant impact on DNAmTL.

**Fig. 3:**
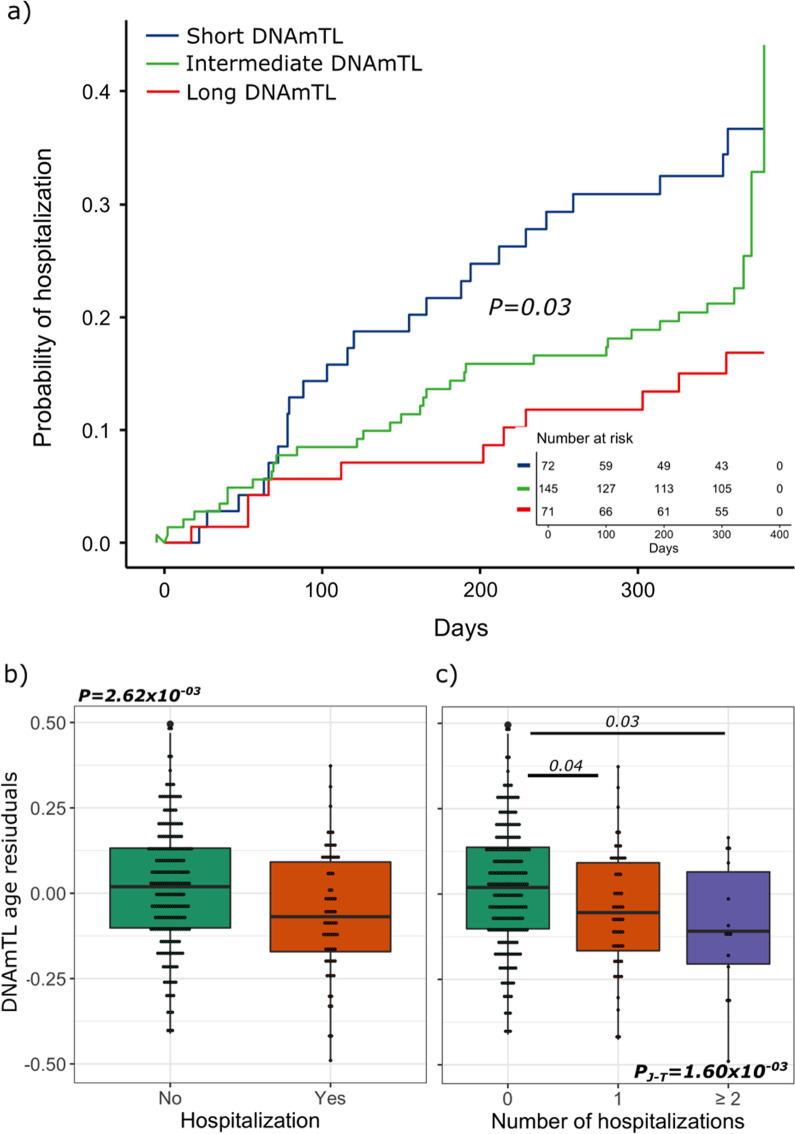
Relationship between hospitalization and DNAmTL age acceleration. **a** *Kaplan–Meier curve* for hospitalization and DNAmTL age acceleration. Axis in (**a**) corresponds to the probability of hospitalization (y-axis) over time (x-axis). The group with the short DNAmTL corresponds to the description “DNAmTL residuals < − 0.12 or 25th percentile” (blue line). DNAmTL residuals (age acceleration) were derived from the regression of DNAmTL on chronological age adjusted for age, sex, body mass index, smoking status and the first five principal components of blood cell proportions. Long DNAmTL corresponds to the description “DNAmTL residuals > 0.13 or 75th percentile” (red line). The description “− 12 = < DNAmTL residuals > = 0.13” (green line) corresponds to the group with an intermediate DNAmTL. The number of patients at risk at each timepoint is provided inside the plots. P-values were generated using a likelihood ratio test (Cox analysis). Boxplots show the DNAmTL age acceleration plotted against hospitalization status (**b**) and number of hospitalizations (**c**). P values at the top of (**c**) correspond to the P-values adjusted for multiple comparison based on the Tukey method. The beginning and the end of the horizontal bars represent the pairwise comparison that corresponds to each P value

Before adjusting for potential confounders, a significant association was found between hospitalization and DNAmTL (*P* = *0.01*). DNAmTL age acceleration was associated with a higher probability of hospitalization over time (Global test *P* = *0.03*); this association was particularly notable in the two extreme groups for DNAmTL age acceleration when they were compared to each other (*P* = *0.01*) (Fig. 3a). Furthermore, hospitalizations and rate of hospitalization (*P* = *5.21* × *10*^*–03*^) were also significantly associated with DNAmTL age acceleration (Fig. 3b, c and Additional file 5). The SGRQ score had a significant association with DNAmTL (*P* = *2.60* × *10*^*–02*^) (Additional file 5), in which a higher score (indicating poor health status) was associated with shorter DNAmTL compared to those with lower scores (indicating good health status).

## Discussion

The most significant finding of our study was that telomeric age acceleration based on blood DNA methylation was associated with increased risk of exacerbations, hospitalizations and poor health status in patients with moderate to severe COPD. Together, these data highlight the importance of replicative senescence in the outcomes of COPD patients and suggest that epigenetic biomarkers may be developed to predict important health events such as exacerbations and hospitalizations in COPD, though additional work will be needed to fully validate this notion.

We have shown previously that short absolute telomere length is associated with increased risk of mortality and rate of exacerbation in COPD [16]. However absolute and relative telomere lengths are influenced by two important factors: telomerase activity and baseline telomere length, both of which may vary between individuals [8], leading to poor signal-to-noise ratio as a biomarker. DNAmTL, on the other hand, is independent of these factors and have a better correlation with chronological age than telomere length itself [11]. Furthermore DNAmTL is able to capture more clinical outcomes compared to aTL [16]. In addition, in vitro assays have shown that DNAmTL reflects cellular replication better than absolute leukocyte telomere length [11], making DNAmTL a more attractive biomarker of replicative senescence than absolute or relative telomere lengths for clinical translation.

Here, we linked DNAmTL to three health outcomes of clinical importance for COPD patients: AECOPD, hospitalizations and SGRQ. First, we found that independent of chronological age, short DNAmTLs are associated with increased risk of exacerbation and hospitalization over time. Second, we demonstrated this relationship to be “dose-dependent” such that DNAmTLs progressively decreased with increasing number of exacerbation events during follow-up. It is important to note that DNAmTL was associated with exacerbations, independent of chronological age and other potential confounders including lung function and smoking status. This observation is consistent with the notion that although telomere shortening is an age-related feature, it is not tantamount to chronological ageing, particularly in the context of COPD. Third, as poor health status often precedes frequent exacerbations and hospitalizations, the inverse relationship between SGRQ and DNAmTL raises the possibility that short telomeres may be apparent earlier in the course of disease.

While it is widely known that COPD is associated with short telomeres [5], the mechanistic link between telomeres and AECOPD is not entirely clear. The epigenetic regulation of telomeric ageing reflects in part cell replication [11]; thus our results support the idea that chronic inflammation in COPD alters the normal life course of cells by accelerating their ageing process and subsequently promoting their arrest and death. It is also possible that AECOPD by itself or the underlying infectious microbial agents may contribute to replicative senescence in COPD. For example, the immune response to infections in COPD patients (i.e.: viral infections) could lead to excessive cellular replication of certain inflammatory cells (e.g. CD8 T cells), leading some (and in particular frequent exacerbators) to demonstrate short telomeres. Although environmental factors such as obesity, and cigarette smoking promote secretion of inflammatory mediators of cellular senescence [25, 26], our analyses adjusted for these key factors, suggesting that there are likely other factors in COPD pathogenesis that are responsible for accelerated telomeric shortening.

Our study had certain limitations. First, we were not able to assess changes in DNAmTL longitudinally. Second, the small number of patients with more than one moderate to severe exacerbations could have impacted our ability to identify additional associations. Third, it remains to be determined whether short DNAmTL is a causal factor for COPD progression or a consequence of the inflammatory and oxidative stress burden of COPD. Fourth, it remains to be determined whether the epigenetic signal in peripheral blood is similar to that in the lung. Last, it is unknown whether certain therapeutics or dietary factors can reverse or halt replicative senescence of COPD.

In summary, short DNAmTL in peripheral blood, reflective of replicative senescence, is associated with increased risk of exacerbation and hospitalization and poor health status in COPD and thus a very promising biomarker to predict poor outcomes in patients with COPD.

## Conclusions

This study provides new evidence to support the notion that the epigenetic regulation of telomeric age in blood plays a key role in the worsening of respiratory symptoms in moderate to severe COPD. Epigenetic biomarkers may be developed to predict important health events such as exacerbations and hospitalizations in COPD, and to identify COPD patients who are at a higher risk of poor outcomes for personalized care.

## Supplementary Information

**Additional file 1: Table S1. **Participants form the Macrolide Azithromycin to Prevent Rapid Worsening of Symptoms Associated with Chronic Obstructive Pulmonary Disease study (MACRO) that were randomized to the placebo arm.

**Additional file 2: Figure S1.** Randomization of the Macrolide Azithromycin to Prevent Rapid Worsening of Symptoms Associated with Chronic Obstructive Pulmonary Disease study (MACRO). Created with BioRender.com.

**Additional file 3: Figure S2.** Linear relationship between DNAmTL (y-axis) and chronological age (x-axis). Green and orange colour represent non-exacerbators, and exacerbators, respectively.

**Additional file 4: Table S2.** Cox analysis: DNAmTL and probability of AECOPD and hospitalization.

**Additional file 5: Table S3.** Clinical features associated with DNAmTL age acceleration.

## Data Availability

The findings of this study are included in the article. Methylation data will be deposited on GEO database after submission. Further inquiries can be directed to the corresponding author.

## Abbreviations

AECOPD: Acute exacerbation COPD
BMI: Body Mass Index
COPD: Chronic obstructive pulmonary disease
DNAmTL: DNA methylation telomere length
FEV_1_: Forced expiratory volume in 1 s
FVC: Forced vital capacity
GOLD: Global initiative for chronic Obstructive Lung Disease
ICS: Inhaled corticosteroids
MACRO: The Macrolide Azithromycin to Prevent Rapid Worsening of Symptoms Associated with Chronic Obstructive Pulmonary Disease study
LABA: Long-acting beta-2 agonists
PC: Principal component
PCA: Principal component analysis
SGRQ: St. George’s Respiratory Questionnaire
