# Supplementary material for: Epigenetic marker of telomeric age is associated with exacerbations and hospitalizations in chronic obstructive pulmonary disease
Source: Respir Res. 2021 Dec 22;22:316. doi: 10.1186/s12931-021-01911-9 (PMC8693486; doi:10.1186/s12931-021-01911-9)
Supplement: Supplementary file 1 — Additional file 1: Table S1. Participants form the Macrolide Azithromycin to Prevent Rapid Worsening of Symptoms Associated with Chronic Obstructive Pulmonary Disease study (MACRO) that were randomized to the placebo arm. [file 12931_2021_1911_MOESM1_ESM.docx]

**Table S1.** Participants form the Macrolide Azithromycin to Prevent Rapid Worsening of Symptoms Associated With Chronic Obstructive Pulmonary Disease study (MACRO) that were randomized to the placebo arm.

|  | Profiled for blood DNA methylation | | **P* |
| --- | --- | --- | --- |
|  | **No** | **Yes** |  |
| n | 281 | 292 |  |
| Age, year | 64.22 ± 8 | 67 ± 8 | 0.0001 |
| Males, No. (%) | 178 (63) | 165 (57) | 0.11 |
| Non-Hispanic whites, No. (%) | 212 (75) | 246 (84) | 0.03 |
| Current smokers, No. (%) | 78 (28) | 53 (18) | 0.01 |
| BMI, kg/m^2^ | 28 ± 7 | 27 ± 6 | 0.44 |
| Post-bronchodilator FVC, % of predicted | 70.75 ± 17.49 | 69.53 ± 17.35 | 0.57 |
| Post-bronchodilator FEV_1_, % of predicted | 40.61 ± 15.72 | 39.3 ± 15.62 | 0.32 |
| FEV_1_/FVC, % | 43.43 ± 13.11 | 42.29 ± 12.05 | 0.32 |
| Inhaled steroid use, No. (%) | 221 (79) | 230 (79) | ~1 |
| ICS/LABA, No. (%) | 157 (58) | 148 (77) | 0.24 |

Continuous variables are described with the mean ± SD. *Differences between the groups’ demographic characteristics were tested using a Wilcoxon test for continuous variables, and a Fisher’s Exact test for count variables. “Post” refers to spirometry tests after bronchodilator use. Denominators used for the percentages (%) correspond to the total number of participants in each group.
