# Supplementary material for: Epigenetic marker of telomeric age is associated with exacerbations and hospitalizations in chronic obstructive pulmonary disease
Source: Respir Res. 2021 Dec 22;22:316. doi: 10.1186/s12931-021-01911-9 (PMC8693486; doi:10.1186/s12931-021-01911-9)
Supplement: Supplementary file 2 — Additional file 2: Figure S1. Randomization of the Macrolide Azithromycin to Prevent Rapid Worsening of Symptoms Associated with Chronic Obstructive Pulmonary Disease study (MACRO). Created with BioRender.com. [file 12931_2021_1911_MOESM2_ESM.docx]

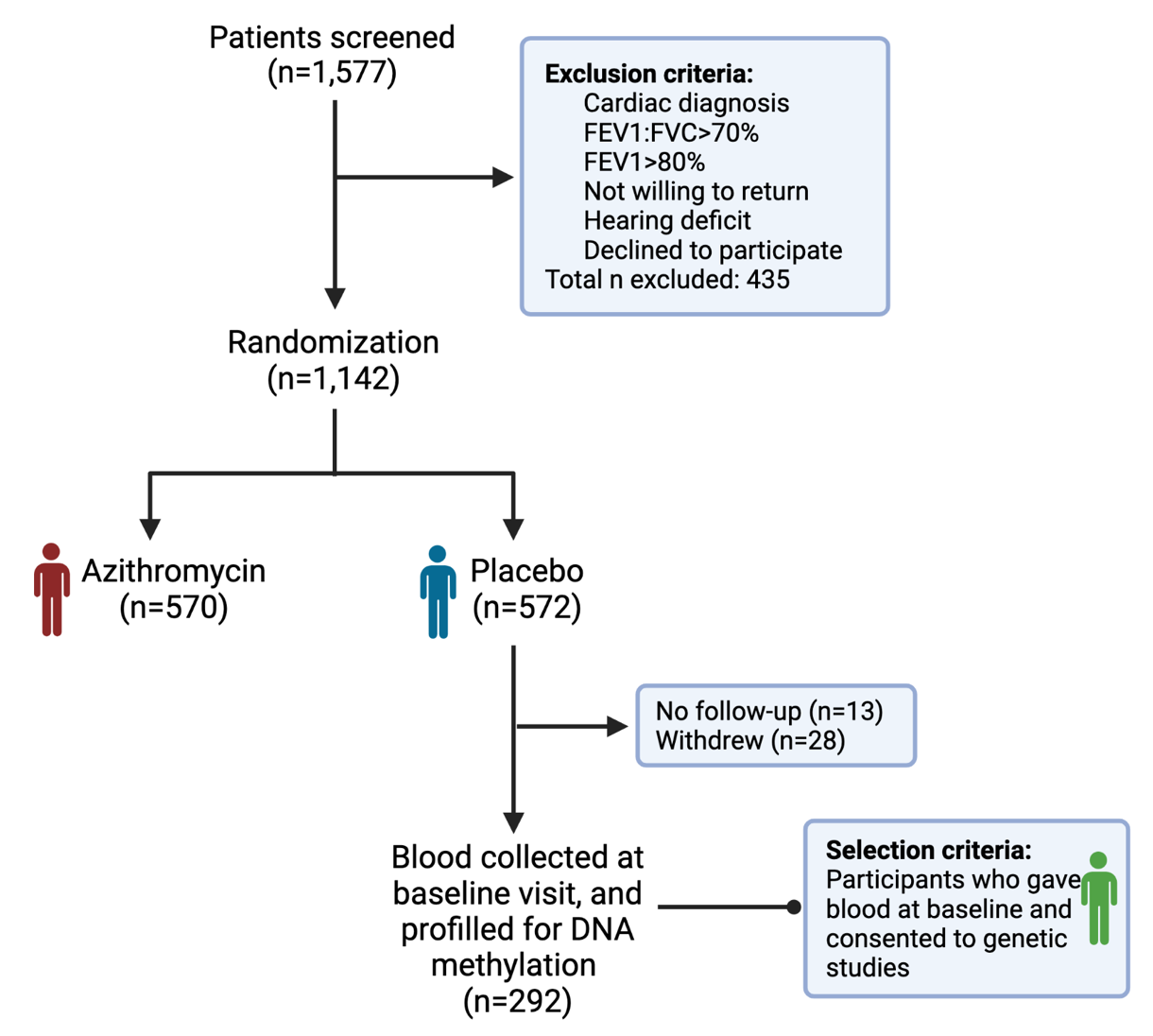


**Figure S1.** Randomization of the Macrolide Azithromycin to Prevent Rapid Worsening of Symptoms Associated With Chronic Obstructive Pulmonary Disease study (MACRO). Created with BioRender.com
