# Supplementary material for: Epigenetic marker of telomeric age is associated with exacerbations and hospitalizations in chronic obstructive pulmonary disease
Source: Respir Res. 2021 Dec 22;22:316. doi: 10.1186/s12931-021-01911-9 (PMC8693486; doi:10.1186/s12931-021-01911-9)
Supplement: Supplementary file 3 — Additional file 3: Figure S2. Linear relationship between DNAmTL (y-axis) and chronological age (x-axis). Green and orange colour represent non-exacerbators, and exacerbators, respectively. [file 12931_2021_1911_MOESM3_ESM.docx]

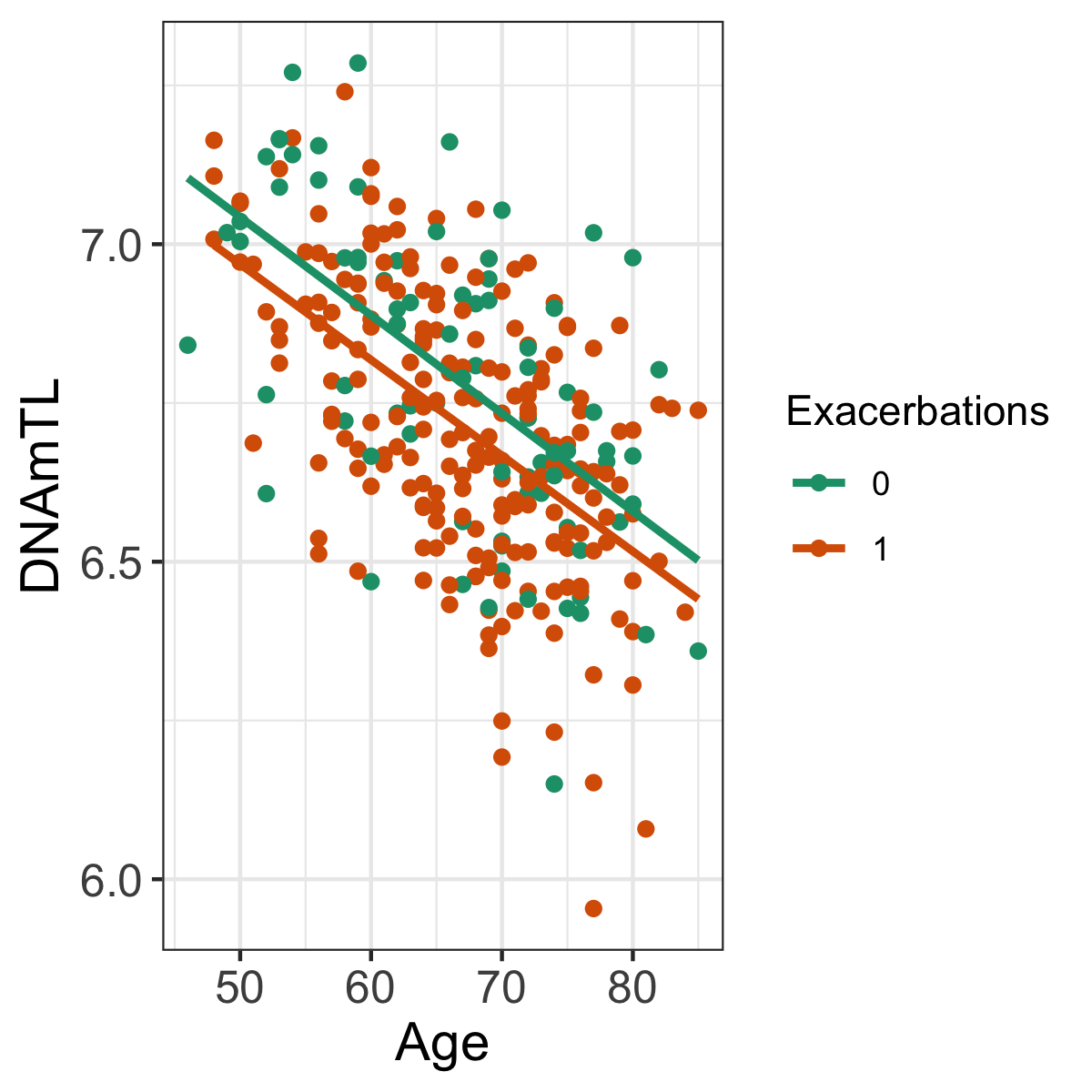


**Figure S2.** Linear relationship between DNAmTL (y-axis) and chronological age (x-axis). Green and orange colour represent non-exacerbators, and exacerbators, respectively.
