# Supplementary material for: Epigenetic marker of telomeric age is associated with exacerbations and hospitalizations in chronic obstructive pulmonary disease
Source: Respir Res. 2021 Dec 22;22:316. doi: 10.1186/s12931-021-01911-9 (PMC8693486; doi:10.1186/s12931-021-01911-9)
Supplement: Supplementary file 4 — Additional file 4: Table S2. Cox analysis: DNAmTL and probability of AECOPD and hospitalization. [file 12931_2021_1911_MOESM4_ESM.docx]

Table S2. Cox analysis: DNAmTL and probability of AECOPD and hospitalization

| **Reference group 🡪** | **DNAmTL ≤ 25^th^ percentile**  **(Short)** | **DNAmTL ≥75^th^ percentile**  **(Long)** |
| --- | --- | --- |
| **AECOPD** | | |
| **≥75th percentile** |  |  |
| Direction | Lower risk |  |
| HR (95% CI) | 0.61 (0.41-0.90) |  |
| *P* | *0.01* |  |
| **25-75th percentile** |  |  |
| Direction | Lower risk | Higher risk |
| HR (95% CI) | 0.71 (0.51-0.97) | 1.15 (0.82-1.63) |
| *P* | *0.03* | *0.42* |
| **≤25th percentile** |  |  |
| Direction |  | High risk |
| HR (95% CI) |  | 1.63 (1.11-2.40) |
| *P* |  | *0.01* |
| **Mild AECOPD** | | |
| **≥75th percentile** |  |  |
| Direction | Lower risk |  |
| HR (95% CI) | 0.60 (0.38-0.94) |  |
| *P* | *0.03* |  |
| **25-75th percentile** |  |  |
| Direction | Lower risk | Higher risk |
| HR (95% CI) | 0.65 (0.45-0.96) | 1.09 (0.73-1.64) |
| *P* | *0.03* | *0.67* |
| **≤25th percentile** |  |  |
| Direction |  | Higher risk |
| HR (95% CI) |  | 1.67 (1.07-2.62) |
| *P* |  | *0.03* |
| **Moderate to severe AECOPD** | | |
| **≥75th percentile** |  |  |
| Direction | Lower risk |  |
| HR (95% CI) | 0.49 (0.27-0.87) |  |
| *P* | *0.02* |  |
| **25-75th percentile** |  |  |
| Direction | Lower risk | Higher risk |
| HR (95% CI) | 0.62 (0.39-0.98) | 1.26 (0.73-2.18) |
| P | *0.04* | *0.41* |
| **≤25th percentile** |  |  |
| Direction |  | Higher risk |
| HR (95% CI) |  | 2.02 (1.13-3.61) |
| *P* |  | *0.02* |
| **Hospitalization** | | |
| **≥75th percentile** |  |  |
| Direction | Lower risk |  |
| HR (95% CI) | 0.40 (0.20-0.83) |  |
| *P* | *0.01* |  |
| **25-75th percentile** |  |  |
| Direction | Lower risk | Higher risk |
| HR (95% CI) | 0.63 (0.37-1.05) | 1.54 (0.77-3.04) |
| *P* | *0.08* | *0.21* |
| **≤25th percentile** |  |  |
| Direction |  | Higher risk |
| HR (95% CI) |  | 2.46 (1.20-5.01) |
| *P* |  | *0.01* |

P-value (P) and hazard ratio (HR) correspond to the Cox regression analyses for total and moderate to severe acute COPD exacerbations (AECOPD), and hospitalizations. Direction: direction of the effect of DNAmTL age residual group compared to the reference groups (**top row**). DNAmTL age residuals correspond to the regression of DNAmTL on chronological age adjusted for sex, body mass index, smoking status and the first five principal components of blood cell proportions. The group with short DNAmTL corresponds to DNAmTL residuals < -0.12 or 25^th^ percentile. Long DNAmTL corresponds to DNAmTL residuals > 0.13 or 75^th^ percentile. The group with intermediate DNAmTL length corresponds to DNAmTL age residuals between the 25^th^ and 75^th^ percentile.
