# Supplementary material for: Epigenetic marker of telomeric age is associated with exacerbations and hospitalizations in chronic obstructive pulmonary disease
Source: Respir Res. 2021 Dec 22;22:316. doi: 10.1186/s12931-021-01911-9 (PMC8693486; doi:10.1186/s12931-021-01911-9)
Supplement: Supplementary file 5 — Additional file 5: Table S3. Clinical features associated with DNAmTL age acceleration. [file 12931_2021_1911_MOESM5_ESM.docx]

Table S3. Clinical features associated with DNAmTL age acceleration

| Variable | Estimate effect on DNAmTL | *P* |
| --- | --- | --- |
| Rate of AECOPD (total) | -0.02 | *1.35x10^-04^* |
| Rate of mild AECOPD | -0.02 | *6.62 x10^-03^* |
| Rate of moderate to severe AECOPD | -0.04 | *9.74x10^-03^* |
| Rate of hospitalization | -0.04 | *5.21x10^-03^* |
| SGRQ total score | -0.002 | *2.60x10^-02^* |

P-value and estimate correspond to linear regression of DNA methylation measurement of telomere length (DNAmTL) on each variable adjusted for age, sex, body mass index, smoking status and the first five principal components of blood cell proportions. Negative estimates indicate that as predictor values increase DNAmTL becomes shorter. AECOPD: acute COPD exacerbations. St. George Respiratory Questionnaire (SGRQ).
